# Phenotypic plasticity in size of ant-domatia

**DOI:** 10.1038/s41598-020-77995-y

**Published:** 2020-12-01

**Authors:** Bertrand Kokolo, Christiane Attéké Nkoulémbéné, Brama Ibrahim, Bertrand M’Batchi, Rumsais Blatrix

**Affiliations:** 1 Laboratoire de Physiologie Animale, Unité de Recherche Agrobiologie, Université des Sciences et Techniques de Masuku (USTM), BP 901, Franceville, Gabon; 2 CEFE, Université de Montpellier – CNRS – EPHE – IRD – Université Paul Valéry Montpellier 3, Montpellier, France

**Keywords:** Ecosystem ecology, Evolutionary ecology, Tropical ecology

## Abstract

Ant-plants produce hollow structures called domatia to host protecting ants. Although size variation in domatia is well documented between related species, intraspecific variation is little explored. The central African ant-plant *Barteria dewevrei* exibits strong variation in domatium size, giving the opportunity to explore the mechanism underlying variation in a mutualistic trait. We showed that domatium size in *Barteria dewevrei* varies between sites. We transplanted individual plants between two sites in Gabon where plants have different domatium sizes. Domatium size of transplanted plants changed, revealing that variation in this mutualistic trait is driven by phenotypic plasticity. The two sites differed in their environmental conditions: highland open savanna on sandy soil vs lowland closed tropical rain forest on sandy-loam soil. However, as stomatal density and δ^13^C of leaves did not differ between sites or between branches produced before and after transplantation, we have no cue on the role of abiotic stress (such as light intensity and water availability) in domatium size variation. As the obligate *Tetraponera* ant symbionts are too large to fit in the small domatia, variation of the mutualistic trait in response to environmental change through phenotypic plasticity may impact this specialized mutualism.

## Introduction

Phenotypic differences between sites may result from differences in genotypes or from phenotypic expression varying according to environmental conditions (abiotic and biotic factors), a phenomenon called phenotypic plasticity^1^. Although phenotypic plasticity is often considered to be adaptive, i.e. phenotype variability is selected in the course of evolution because it optimizes fitness in a range of environmental conditions, it must be kept in mind that phenotypic plasticity may result from environmental constraints limiting trait expression^2^. Variation in functional traits involved in mutualistic interactions is likely to affect costs and benefits of mutualism. Characterizing the process (genotypic variation or phenotypic plasticity) driving such variation should help in understanding patterns in mutualistic interactions. Ant-plants can be used as models in this respect because they bear conspicuous architectural traits for hosting ants, traits that are involved in mutualistic interactions and that can be measured easily.

Ant-plants are defined as plants providing ants with nesting sites in the form of hollow structures called domatia, such as modified stems, leaves, stipules, petioles, etc. Although domatia were first thought to be induced by ants^3^, there is now extensive evidence that most are spontaneously produced and many ant-plant interactions are mutualistic^4^. The ants benefit the plant mainly by deterring herbivores and providing nutrients^5^. Variation in domatium presence and size has been documented among groups of related species (typically within genera), and has given insights into the role of environmental conditions in mutualism state. For instance, a pattern that seems general is the loss of domatia in species living at high elevation^6–8^. The underlying hypothesis is that herbivore pressure and ant diversity both decline with increasing elevation, reducing the benefits, but not the costs, of bearing mutualistic traits^9^. In contrast, intra-specific variation in domatium traits has been little explored.

Herbivory was shown to trigger production of larger domatia. In *Cordia nodosa*, plants growing in herbivore exclusion cages produced smaller domatia than plants under herbivore pressure^10^, suggesting adaptive phenotypic plasticity. Similarly, *Acacia drepanolobium* produces fewer and smaller domatia in large mammalian-herbivore exclusion plots^11,12^. Moreover, artificial shoot clipping simulating herbivory on herbivore-excluded plants induced rapid and localized increase in domatium length, demonstrating phenotypic plasticity of domatium size at the branch level^11^. Similarly, *Acacia drepanolobium* trees pruned by the ant *Crematogaster nigriceps* are characterised by larger and denser domatia^13^. *Humboldtia brunonis* is one of the rare ant-plant species in which both domatia-bearing and domatia-free individuals occur^14^. In this species, the proportion of domatia-bearing plants varies geographically and is higher in sites with stronger herbivore pressure^15^. Ants that live on plants sometimes cut floral buds, which increases vegetative growth, and eventually benefits the ant colony. In the plant *Hirtella physophora*, such a castration process leads to smaller domatia^16^, suggesting a phenomenon of retaliation against cheating ants. Altogether, these studies show that physical damages to the plant can induce intra-specific variation in domatium state.

In *Barteria dewevrei* (Passifloraceae), a small tree (up to 10 m) endemic to central Africa, domatium state ranges from non-swollen branches (absence of domatium) to branches swollen and hollow throughout their length with inner cavities up to 8.5 mm in diameter. In Gabon, trees with larger domatia are found in lowland tropical rain forest and are occupied by large *Tetraponera* ant species (8–10 mm) that are obligate inhabitants of *Barteria* plants, whereas trees with smaller domatia are found in the savannah ecosystem of the Batéké Plateau and are occupied by small *Crematogaster* ants (< 4 mm)^8,17^. Thus, this plant species is well suited for investigating the determinism of spatial variation in a mutualistic trait. The aim of the present study was to determine whether between-site differences in domatium diameter in this species is the result of phenotypic plasticity or of differences in genotypes.

To test for phenotypic plasticity of domatium size, we measured domatium size on *B. dewevrei* individuals transplanted reciprocally between two sites with contrasting domatium sizes. In case of phenotypic plasticity, we expected domatium size of transplanted trees to change. Transplanted trees did not have ants, so that domatium size variation could not be attributed to a direct effect of ants. The two sites had contrasted environmental conditions and corresponded to lowland tropical rain forest and savannah on sandy slope. Differential soil drainage and canopy cover may induce contrasted water availability and light intensity between these two ecosystems. Stress related to these two parameters may affect plant growth, and thus domatium size, and could be a proximate mechanism driving domatium size variation. Stomatal density and δ^13^C were measured in leaves to test whether transplanted trees experienced contrasted conditions of water stress in these two ecosystems. We expected stomatal density and δ^13^C to increase in trees transplanted to the savannah site because stomatal density was shown to increase with light intensity^18^ and δ^13^C was shown to decrease with water availability^19^, although causes of variation in δ^13^C are complex^20^.

## Material and methods

**Figure 1:**
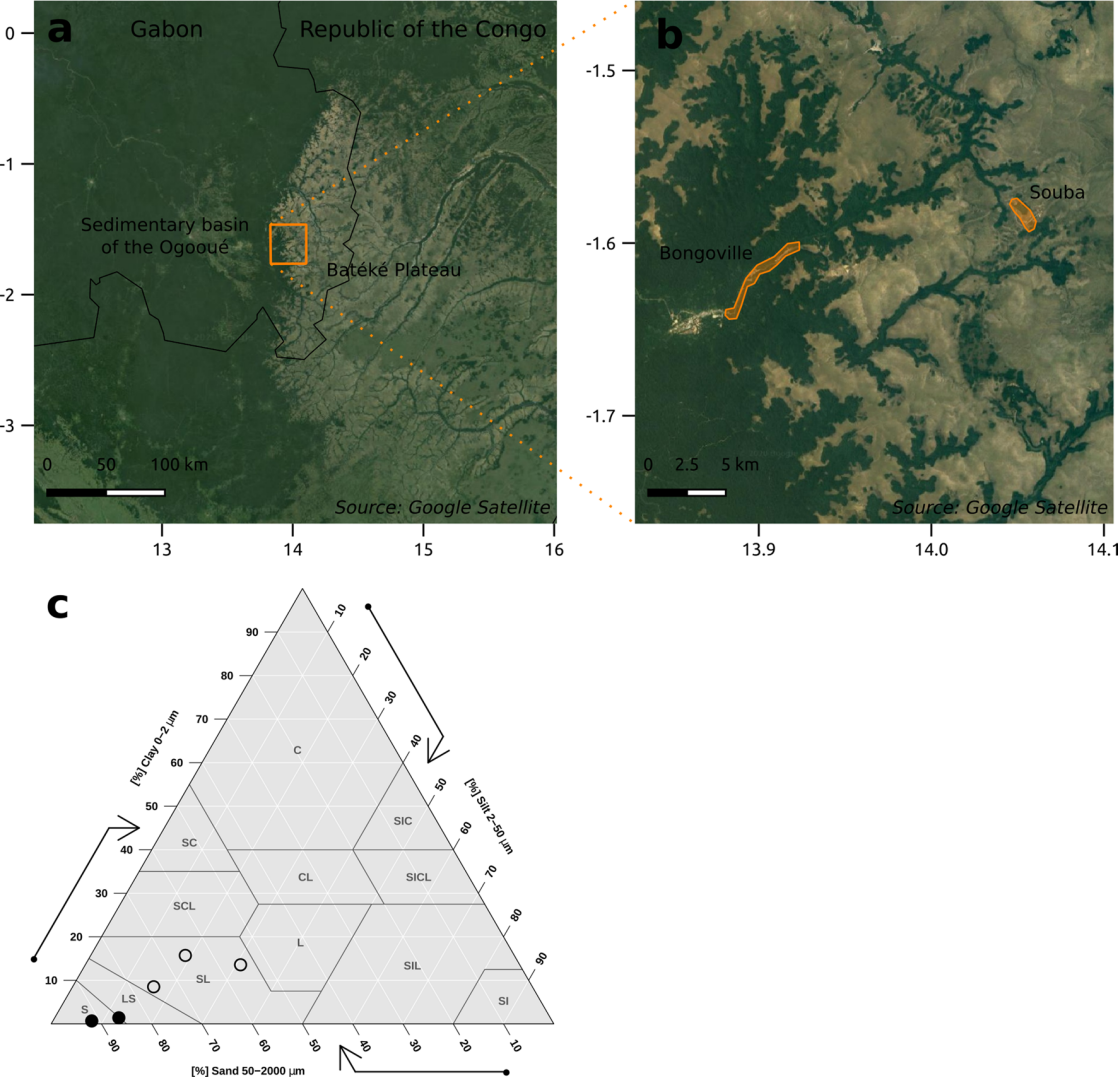
Contrasted environmental conditions in the two study sites in Gabon, Bongoville and Souba. (**a**) Regional positioning of the study area, at the interface of the sedimentary basin of the headwaters of the Ogooué and the Batéké Plateau. (**b**) Local positioning of the study sites, in the lowland tropical rain forest for Bongoville and in the highland savanna for Souba. All maps were generated by the authors with QGIS 3.10 (www.qgis.org) using Google Satellite images as background satellite data. (**c**) Texture of Bongoville and Souba soils (empty and plain circles respectively) represented on a texture triangle using the USDA classification of soils.

To test for the effect of environmental conditions on domatium size, we choose study sites that differed both in domatium size and environmental conditions. Reciprocal transplantation was conducted between two sites in south-eastern Gabon: Souba (1.58° S 14.05° E, 550 m asl) and Bongoville (1.62° S 13.90° E, 360 m asl) (Fig. 1a,b). Although the two sites are only 17 km apart, they have contrasting environmental conditions. Bongoville lies on the sedimentary basin of the Ogooué river headwaters, characterized by sandy clay poor soils on gentle slope^21^. Souba is located on the Batéké Plateau, a Tertiary continental shelf characterized by sandy poor soils on steep slope, subject to leaching^21^. To confirm that the pedological conditions of these two geological units apply to our study sites, we analysed two and three soil samples (top 10 cm of the soil column) from Souba and Bongoville respectively. Soil texture, pH (in water) and organic matter content were analysed and measured in the *Laboratoire d'Analyses Agricoles Teyssier* (Bourdeaux, France). Soil in Bongoville and Souba was acidic (pH 4.5–5.2) and contained 2.5–3.2% of organic matter. Soil in Bongoville contained more clay and less sand than in Souba (Fig. 1c). Soil was classified as sandy in Souba and sandy loam in Bongoville, according to the USDA system^22^, confirming a contrasted soil texture between the two sites. The two sites also differ in the ecosystems they harbour: in Bongoville a typical lowland tropical rain forest with a closed canopy; in Souba an open savannah typical of the Batéké Plateau (differences are visible on satellite images on Fig. 1a,b). *Barteria dewevrei* is a small, fast-growing pioneer tree, colonizing forest edges. In Bongoville it receives some shade from the adjacent high canopy, whereas in Souba it is more exposed to direct sunlight. Geomorphological, pedological and ecological differences between the two sites suggest that *B. dewevrei* should experience higher insolation and water stress in Souba than in Bongoville. These environmental constraints, if affecting plant growth, may influence domatium size.

*Barteria* trees are usually colonized by ants once they reach a height of more than 1 m. For the transplantation experiment we selected unoccupied *Barteria dewevrei* seedlings 0.3 to 1 m in height so that phenotypic changes, if any, could not be attributed to the direct effect of local ants colonizing the trees. Such a putative effect is unlikely anyway because domatia of *Barteria* have been reported to reach their final size before being colonized by ants^23^.

Seventeen trees were transplanted from Bongoville to Souba, out of which 10 survived. Twenty-two trees were transplanted from Souba to Bongoville, out of which 13 survived. The control treatment consisted in transplanting trees within each site. This treatment allowed to control for a potential effect of transplantation (uprooting, transport and replanting) on phenotypic traits. Eleven control trees were transplanted within Bongoville, out of which five survived. Nine control trees were transplanted within Souba, out of which four survived. Size of selected trees before transplantation and tree survival did not differ among the four groups of transplanted trees (two groups transplanted between sites, and two control groups transplanted within sites) (respectively, Kruskal–Wallis: statistic = 2.7, *p* = 0.43, Chi-squared test: Chi^2^ = 1.0, *p* = 0.71). *Barteria dewevrei* trees have a single vertical (orthotropic) trunk and unforked lateral (plagiotropic) branches that are hollow throughout their length, each forming a domatium. For each plant, we measured the diameter of the cavity on a recently developed lateral branch before transplantation and then on a new recently developed lateral branch 12 months after transplantation. We ensured that lateral branches measured after transplantation had not started to develop before transplantation, which was possible because *Barteria* trees are fast-growing.

Stomatal density and δ^13^C were measured to confirm or not that trees experienced more stressful environmental conditions in Souba than in Bongoville. Stomatal density was measured on the same trees, before and 12 months after transplantation. Each measurement was based on three fully developed leaves collected on recently developed branches. A thin layer of clear nail varnish was applied on the abaxial side of the leaf, left to dry for 20 min., detached using adhesive transparent tape and stuck onto a microscope slide. Three slides were prepared for each leaf. Stomata were counted using a microscope at × 400 magnification on a total leaf surface of 1.43 mm^2^ for each tree. We measured δ^13^C on the same trees, 12 months after transplantation. Samples of fully developed leaves were collected on recently developed branches and dried with silica gel immediately upon collection. Isotopic abundance (C) was measured with an elemental analyser (EuroVector, Pavia, Italy) connected to an isotopic mass spectrometer (Isoprime, Elementar, Stockport, UK) at the stable isotopes platform of the Biochemistry & Plant Molecular Physiology research unit (Montpellier, France).

Measurements of domatium diameter and stomatal density taken before and after transplantation (either between or within sites) were compared with Wilcoxon signed rank tests for paired samples as trees were individually labelled. Given the low sample size for control trees surviving after within site transplantations (five and four in Bongoville and Souba respectively), testing the effect of within site transplantation with tests for paired samples could suffer from low statistical power, and thus, high risk of type II error. The risk of failing to reject the null hypothesis when it is false (type II error) should be as low as possible for a control experiment such as the within-site transplantation. Thus, we also performed Mann–Whitney U-tests for independent samples to compare, within each site, measurements taken before transplantation (including trees that did not survive transplantation) with measurements after transplantation (only surviving trees), and provided the corresponding figures and test values as Supplementary Information. For all comparisons, significance was the same for both types of tests. Measurements of δ^13^C were performed after transplantation only, and thus, were compared with Mann–Whitney U-tests. p values were adjusted using Holm's method in case of multiple comparisons.

## Results

**Figure 2:**
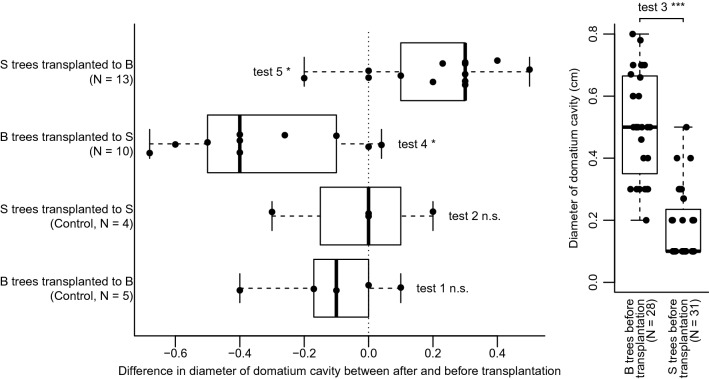
Variation in size of the cavity of *Barteria dewevrei* domatia in Souba and Bongoville, Gabon, before and 12 months after transplantation. Thick lines represent median, boxes represent interquartile range, and whiskers extend to the data extremes. B: Bongoville, S: Souba, N: sample size, *n.s.* non significant, *p < 0.05, ***p < 0.001.

**Figure 3:**
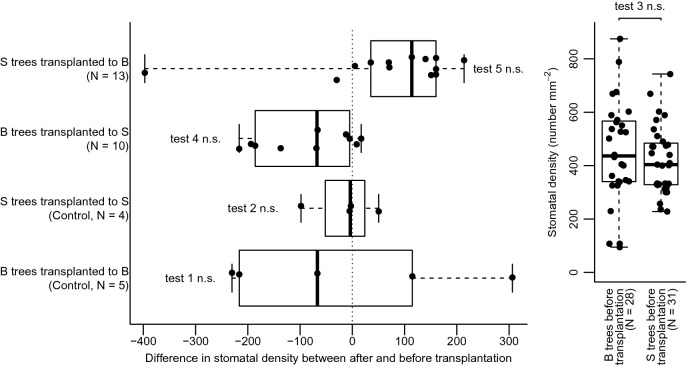
Variation in stomatal density on the abaxial side of *Barteria dewevrei* leaves in Souba and Bongoville, Gabon, before and 12 months after transplantation. Thick lines represent median, boxes represent interquartile range, and whiskers extend to the data extremes. B: Bongoville, S: Souba, N: sample size, *n.s.* non significant.

Transplantation within sites (control treatment) had no significant effect on domatium size (Wilcoxon signed rank tests for paired samples, Bongoville: W = 8.5, *p*_adj_ = 0.54, Souba: W = 2, *p*_adj_ = 1, Fig. 2, tests 1 and 2, respectively; for Mann–Whitney U-tests for independent samples see Supplementary Fig. S1) and stomatal density (Bongoville: W = 8, *p*_adj_ = 1, Souba: W = 7, *p*_adj_ = 1, Fig. 3, tests 1 and 2, respectively; for Mann–Whitney U-tests for independent samples see Supplementary Fig. S2).

**Figure 4:**
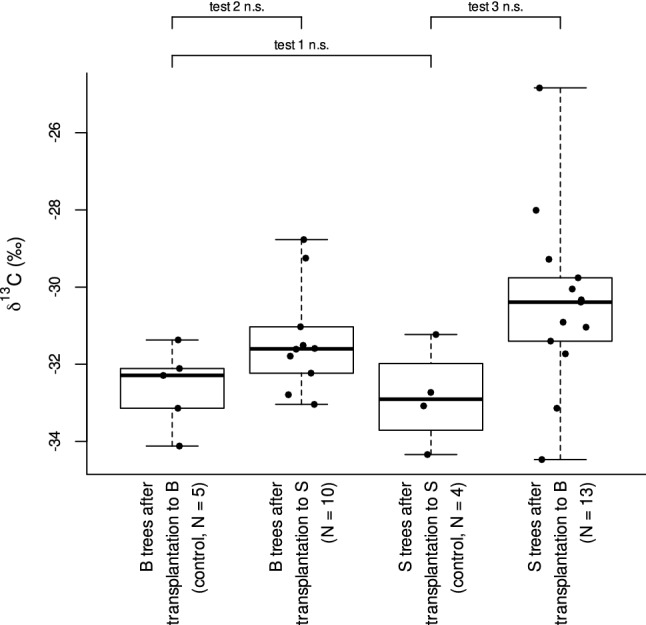
Value of δ^13^C of *Barteria dewevrei* leaves in Souba and Bongoville, Gabon, after transplantation. Horizontal lines represent median, boxes represent interquartile range, and whiskers extend to the data extremes. B: Bongoville, S: Souba, N: sample size, *n.s.* non significant.

Before transplantation, domatia of plants in Souba were smaller in diameter than those of plants in Bongoville (Mann–Whitney U-tests for independent samples, U = 822, *p*_adj_ < 0.0001, Fig. 2, test 3). Stomatal density was not significantly different between the two sites before transplantation (U = 501, *p*_adj_ = 0.94, Fig. 3, test 3). As we did not measure δ^13^C before transplantation, we tested for difference between the two sites by comparing δ^13^C in control trees after transplantation (within their original site). These values did not differ significantly (U = 11, *p*_adj_ = 0.90, Fig. 4, test 1).

Twelve months after transplantation from Bongoville to Souba, trees produced domatia with a smaller diameter than in their original site (W = 44, *p*_adj_ = 0.038, Fig. 2, test 4; for Mann–Whitney U-tests for independent samples see Supplementary Fig. S1). Conversely, trees transplanted from Souba to Bongoville produced domatia with a larger diameter than in their original site (W = 3, *p*_adj_ = 0.034, Fig. 2, test 5; for Mann–Whitney U-tests for independent samples see Supplementary Fig. S1). Transplantation had no significant effect on stomatal density (Bongoville to Souba: W = 49, *p*_adj_ = 0.14; Souba to Bongoville: W = 15, *p*_adj_ = 0.14, Fig. 3, tests 4 and 5, respectively; for Mann–Whitney U-tests for independent samples see Supplementary Fig. S2). Although δ^13^C tended to be higher for transplanted trees than for trees transplanted within their original site, differences were not significant (Bongoville to Souba: U = 12, *p*_adj_ = 0.26; Souba to Bongoville: U = 9, *p*_adj_ = 0.18, Fig. 4, tests 2 and 3, respectively).

In total, transplantation induced a 46% mortality rate. Trees that did not survive transplantation from Bongoville to Souba had a smaller height than the surviving ones (Mann–Whitney U-tests: U = 61, *p* = 0.015). No difference was detected for the three other groups of transplanted trees.

## Discussion

Our transplantation experiment demonstrated a predominant role of phenotypic plasticity in explaining the smaller diameter of domatia in Souba than in Bongoville: Souba plants grew larger domatia after transplantation to Bongoville and Bongoville plants grew smaller domatia after transplantation to Souba. This difference was not due to the stress provoked by transplantation because domatium size did not change in plants transplanted within the same site.

By definition, proximate causes of phenotypic plasticity are environmental, either abiotic or biotic. In our study we did not detect any significant variation of stomatal density or of δ^13^C in leaves of *B. dewevrei*, either before and after transplantation, or between the two sites before transplantation. Stress putatively induced by transplantation (uprooting, transport and replanting) should be gone by twelve months, and should not have influenced measures of stomatal density and δ^13^C. Survival after transplantation could have revealed different growth conditions between sites, but it did not differ among the four groups of transplanted trees. Although the two study sites differed markedly in their environmental conditions (see “Material and methods” section), potential stress induced by water availability and insolation has not translated into detectable differences in stomatal density or in δ^13^C. First, sample size was small, preventing the detection of subtle differences. The sample size needed to detect differences depends on the magnitude of the differences. The magnitude of change of domatium size after transplantation was large enough to detect differences in domatium size despite the small sample size. This may not be the case for stomatal density and δ^13^C. Second, given the complexity of the relationship between stress and these two plant traits, stress experienced by transplanted trees may not have induced changes in stomatal density and δ^13^C. Thus, we are left with no cue on the role of stress as an abiotic proximate factor accounting for the production of domatia smaller in Souba than in Bongoville. As we did not find ants colonizing the experimental trees while assessing domatium size, the occurrence of different ant species between the two sites could not have been the proximate biotic factor that triggered domatium size variation. In previous cases of intraspecific variation of domatium size, physical damage to the plant was the trigger, either as the result of herbivore attack^10,11^, or as the result of symbiotic ants pruning their host plant^13,16^. The trees used in our experiment may have been particularly exposed to herbivory because of the absence of symbiotic ants. Thus, herbivory may have been a proximate cause of phenotypic plasticity in *B. dewevrei*. We noticed herbivory on transplanted trees in our experiment, but we unfortunately did not measure it.

Previous investigation in Gabon showed that *Barteria* trees were occupied by *Crematogaster* sp. (less than 4 mm long) in the savannah environment of the Batéké Plateau and by *Tetraponera* ants (8–10 mm long) in the lowland rain forest^8,17^, a pattern that we confirmed in our two study sites by checking mature trees (qualitative assessment). The match between domatium size and ant species could result from a mere filtering mechanism: *Tetraponera* ants are too large to colonize trees with small domatia but may outcompete *Cermatogaster* in trees with large domatia. Thus, proximate factors of domatium size variation may have cascading effects on the identity of the ant symbiont. Alternatively, differences in ant and herbivore communities between the two sites may be the ultimate cause of domatium size variation. As ant communities are different between tropical forests and savannas^24,25^, spatial distribution of the two ant species or environmentally driven variation in their protection efficiency may have driven selection for phenotypic plasticity of domatium size. Production of large domatia may be maintained in tropical rainforest because *Tetraponera* ants (the larger of the two symbionts) are specifically equipped for protection against large mammals^26^ such as monkeys^27^, that are common in this forest type. However, the plant may benefit from investing less in domatia in environments where the larger symbiotic ant is absent or inefficient. *Tetraponera latifrons* is a tropical rain forest specialist that may be unable to survive or protect its host plant in high-insolation savanna environments, where it would experience (in domatia and on the plant’s surface) a much broader range of temperatures than in tropical rain forest. *Tetraponera* associates of *Barteria* may not be able to withstand temperatures reached under the high insolation typical of open tropical savannas, as suggested by the observation that when the tropical rain forest specialist tree *Barteria fistulosa* occurs in open, sunny clearings it is occupied by *Crematogaster* instead of *Tetraponera* ants (Doyle McKey, personal communication), although size of domatia is suitable for *Tetraponera* ants. The thermal optimal range of symbiotic ants was shown to constrain protection efficiency of the ant-plant *Acacia* (*Vachellia*) *drepanolobium*^28^, although in this case, protection efficiency decreased with temperature.

Our study is the first to demonstrate that phenotypic plasticity explains between-site differences in domatium size. In contrast, the African ant-plant *Leonardoxa africana* has a subspecies that consistently lacks domatia whether it occurs in submontane forest on hilltops or in lowlands^29^, and myrmecophytic *Neonauclea* produce domatia whatever the elevation^30^. The extent to which domatium size or occurrence in ant-plants exhibits plasticity remains to be investigated. Evolutionary loss of ant-domatia often follows colonization of a new environment such as high-elevation sites^6,8^. A next step would be to test whether these high-elevation taxa that lack domatia retained the ability to produce domatia in lowland conditions. Although field transplantation of plants (and trees in particular) can be technically challenging, it is a powerful approach to decipher potential causes of domatium loss.

## Supplementary information

Supplementary Information.

## Data Availability

The datasets generated during and/or analysed during the current study are available from the corresponding author on reasonable request.
