# Supplementary material for: Phenotypic plasticity in size of ant-domatia
Source: Sci Rep. 2020 Dec 1;10:20948. doi: 10.1038/s41598-020-77995-y (PMC7708978; doi:10.1038/s41598-020-77995-y)

Supplementary information associated with:
Kokolo B., Attéké Nkoulémbéné C., Ibrahim B., M'Batchi B., Blatrix R. Phenotypic plasticity in size of ant–domatia. *Scientific Reports* (2020).

Supplementary Fig. S1: Size of the cavity of *Barteria dewevrei* domatia in Souba and Bongoville, Gabon, before and 12 months after transplantation. Horizontal lines represent median, boxes represent interquartile range, and whiskers extend to the data extremes. Test values refer to Mann-Whitney U-tests for independent samples. B: Bongoville, S: Souba, N: sample size, n.s.: non significant, U: test statistic, p_{adj}: p-value adjusted for multiple comparisons using Holm's method.

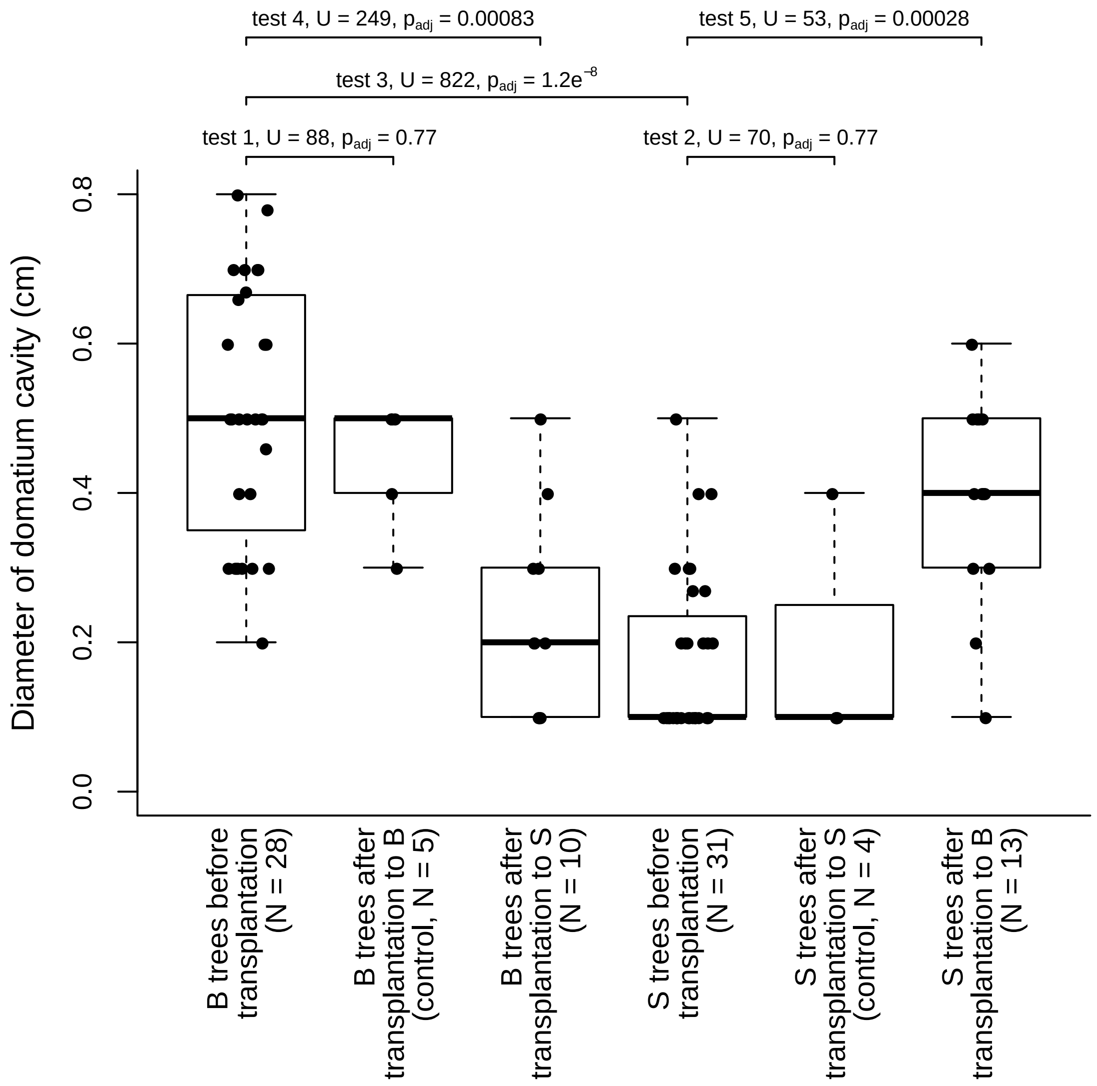

Supplementary Fig. S2: Stomatal density on the abaxial side of *Barteria dewevrei* leaves in Souba and Bongoville, Gabon, before and 12 months after transplantation. Horizontal lines represent median, boxes represent interquartile range, and whiskers extend to the data extremes. Test values refer to Mann-Whitney U-tests for independent samples. B: Bongoville, S: Souba, N: sample size, n.s.: non significant, U: test statistic, p_{adj}: p-value adjusted for multiple comparisons using Holm's method.

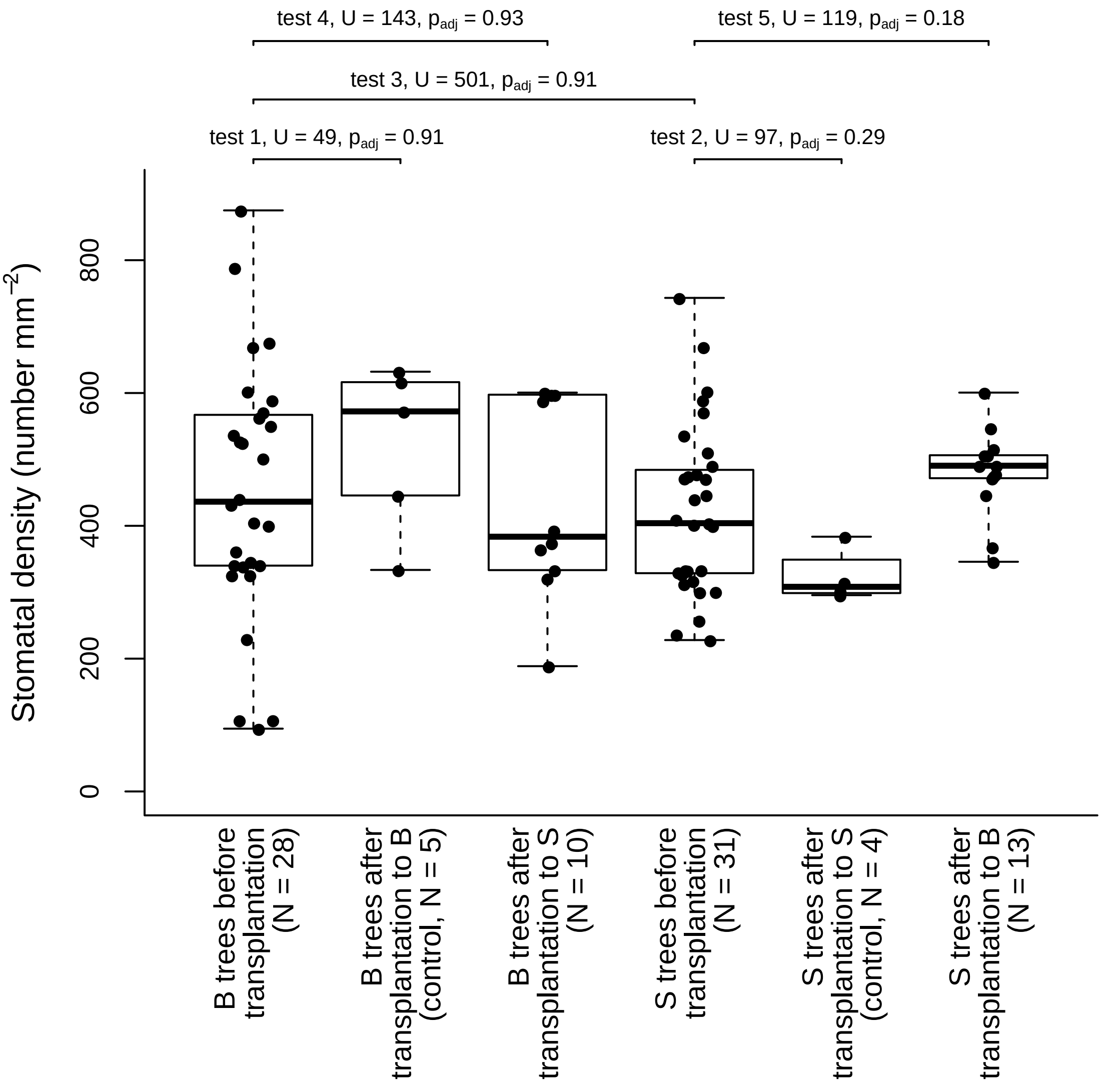

Supplement: Supplementary file 1 — Supplementary Information. [file 41598_2020_77995_MOESM1_ESM.pdf]
